# Anthropometric Indicators as Predictors of Mortality in Early Life Among Low Birthweight Indian Infants

**DOI:** 10.3389/fnut.2022.884207

**Published:** 2022-07-12

**Authors:** Tarun Shankar Choudhary, Mohan Kumar, Bireshwar Sinha, Saijuddin Shaikh, Sarmila Mazumder, Sunita Taneja, Nita Bhandari

**Affiliations:** ^1^Knowledge Integration and Transformation Platform at Centre for Health Research and Development, Society for Applied Studies, New Delhi, India; ^2^Department of Global Public Health and Primary Care, University of Bergen, Bergen, Norway; ^3^Centre for Health Research and Development, Society for Applied Studies, New Delhi, India; ^4^DBT/Wellcome India Alliance Clinical and Public Health Fellow, Hyderabad, India

**Keywords:** anthropometry, low birthweight (LBW), mortality, India, infant

## Abstract

**Background:**

Low birthweight (LBW) babies (<2.5 kg) are at higher risk of mortality and weight for height *z* score is currently recommended for identifying infants at risk of mortality.

**Objective:**

To compare different anthropometric measures at 28-day of age in a cohort of LBW Indian infants for predicting mortality between 28-day and 180-day of age.

**Methods:**

We used data from an individually randomized controlled trial of LBW infants weighing between 1,500 and 2,250 g. Sensitivity, specificity, positive, and negative likelihood ratios, positive and negative predictive values, and area under receiver operating characteristics curves (AUC) were used to estimate the discrimination of mortality risk. The Cox regression was used to estimate hazard ratios and population attributable fraction for each anthropometric indicator. These estimates were calculated for individual as well as combinations of anthropometric indicators at the cut-off of –2 and –3 *SD* of the WHO 2006 growth standards.

**Results:**

Severe underweight (weight-for-age z-scores [WAZ] < –3) had a sensitivity of 75.0%, specificity of 68.0% with an AUC of 0.72. The risk of death was higher (*HR* 6.18; 95% *CI* 4.29–8.90) with a population attributable fraction of 0.63 (95% *CI* 0.52–0.72) for infants severely underweight at 28-day of age. Combination of different anthropometric measures did not perform better than individual measures.

**Conclusion:**

Severe underweight (WAZ < –3) better discriminated deaths among LBW infants < 6 months of age. It can be considered for diagnosis of nutritionally at-risk infants in this age group.

**Clinical Trial Registration:**

[ClinicalTrials.gov], identifier [NCT02653534].

## Introduction

Low birthweight (LBW, birthweight < 2,500 g) is a global public health problem and these infants are at a high risk of mortality (1). Global estimates suggest that in 2015, 20.5 million infants or 14.6% of all live births were born LBW, of which 7.8 million (39%) were in India (2). More than four-fifth of all neonatal deaths in India are among LBW neonates (3–6). Studies have shown that the adjusted risk of neonatal and post-neonatal (28–364 days) mortality in LBW infants is 25, and 7 times higher, respectively, compared to infants with birthweight ≥ 2,500 g (7–9). Therefore, identification of predictors of mortality among LBW infants is critical for timely management.

Infant anthropometric indicators including weight and length are frequently measured as a part of routine postnatal care and to assess nutritional status in public health programs. Studies have shown association between anthropometric indicators and infant mortality (10). Parameters like weight-for-age z-scores (WAZ), weight-for-length z-scores (WLZ), length-for-age z-scores (LAZ), and mid-upper-arm circumference (MUAC) have been used as indicators to identify children with high risk of mortality (11–15). The World Health Organization (WHO) recommends a WLZ < –3 SD for all under-five children (including 0–5 months) and a cut-off of MUAC < 115 mm for children aged 6–59 months to identify severe acute malnutrition, given the high risk of mortality in these children (16–18). Nonetheless, WLZ cannot be calculated using the WHO 2006 growth standards for infants shorter than 45 cm and there are concerns about accuracy of length measurement especially in early infancy (15, 19, 20). Recent evidence suggests that WAZ is better at identifying infants at high risk of death or morbidities compared to WLZ, which had poor reliability and poor prognostic ability (10, 21). Currently, there is no clear consensus on which anthropometric indicator can best predict mortality in the first 6 months of life. Moreover, data specific to the group of LBW children are limited. The aim of our study was to compare WLZ, LAZ, and WAZ measured at 1 month of age, individually and in different combinations, as predictors for the risk of death between 1 and 6 months of age in a cohort of LBW Indian infants.

## Subjects and Methods

### Study Description

We present findings from secondary analysis of an individually randomized controlled trial conducted to assess the impact of supporting mothers in providing kangaroo mother care in community settings on mortality in neonatal period and early infancy (22). The trial was conducted in Faridabad and Palwal districts of Haryana, India between July 2015 to October 2018. Detailed study methodology has been published earlier (22, 23). We enrolled infants within 72 h of birth if they weighed between 1,500 and 2,250 g. We excluded infants who were unable to feed, had difficulty in breathing, had less than normal movements, had gross congenital malformations, kangaroo mother care was initiated in hospital, or whose caregivers intended to move away over the next 6 months or refused participation. Written informed consent was obtained from the infant’s parents at enrolment. The primary trial was approved by the ethics committee of Society for Applied Studies in India, the Regional Committee for Medical and Health Research Ethics in Norway, and World Health Organization, Geneva. The trial is registered at ClinicalTrials.gov (NCT02653534).

### Anthropometric Measurements

Trained interviewers collected household socioeconomic and demographic information at enrollment. Anthropometry was assessed at enrollment, 28-day, 90-day, and 180-day visits by an independent team. Weight to the nearest 10 g was measured using a digital hanging weighing scale (AWS-SR-20; American Weigh Scale, Cumming, GA, United States), calibrated every morning using standard weights. Recumbent length was measured to the nearest 0.1 cm using an Infantometer (model 417; seca GmbH & Co KG). Outcome assessment teams were trained and standardized before study initiation as per WHO guidelines (24, 25). Retraining exercises were conducted every 6 months (26, 27). A team of two workers took two measurements for length and weight and the mean value was used for all analyses.

### Inclusion and Exclusion Criteria

We included infants for whom both, anthropometric assessment at 28-day visit and survival status at 6 months were available. We excluded infants with WAZ < –5 and > + 5, LAZ < –6 and > + 6, and WLZ z scores < –6 and > + 5 were excluded from the analysis (25).

### Statistical Analysis

Categorical and continuous variables were presented as n (%) and mean ± *SD*, respectively. WLZ, LAZ, and WLZ scores were calculated based on the WHO 2006 growth standards using “*zscore06”* (28). Underweight, stunted, and wasted were defined as WAZ < –2, LAZ < –2, and WLZ < –2 and severe underweight, severe stunted, and severe wasted were identified as WAZ < –3, LAZ < –3 and WLZ < –3, respectively (20). We also generated combinations for these, i.e., concurrent wasting and stunting, concurrent wasting and underweight, concurrent stunting and underweight, concurrent severe wasting and severe stunting, concurrent severe wasting and severe underweight, and concurrent severe stunting and severe underweight. We estimated sensitivity, specificity, positive, and negative likelihood ratios, positive and negative predictive values, and area under receiver operating characteristics curves (AUC) for different anthropometric indicators at 28-day for estimating the ability to predict death between 28-day and 180-day of life. We used “*roccomp”* to compare AUC for different anthropometric indicators. We also calculated the hazard ratio (HR) [95% confidence intervals (*CI*)], Population Attributable Fractions (95% *CI*) and estimated the model fit using Harrell’s C concordance for death between 28-day and 180-day for stunting, wasting, underweight, severe stunting, severe wasting, severe underweight, concurrent wasting and stunting, concurrent wasting and underweight, concurrent stunting and underweight, concurrent severe wasting and severe stunting, concurrent severe wasting and severe underweight, and concurrent severe stunting and severe underweight. All analyses were conducted at the cut-off of -2 *SD* and -3 *SD*. The analyses were adjusted for the intervention and cluster robust standard errors were used to account for clustering of deaths in households with multiple births and for infants enrolled from the same household. A *p*-value < 0.05 was considered statistically significant. All analyses were conducted in Stata 17 (29).

## Results

**FIGURE 1:**
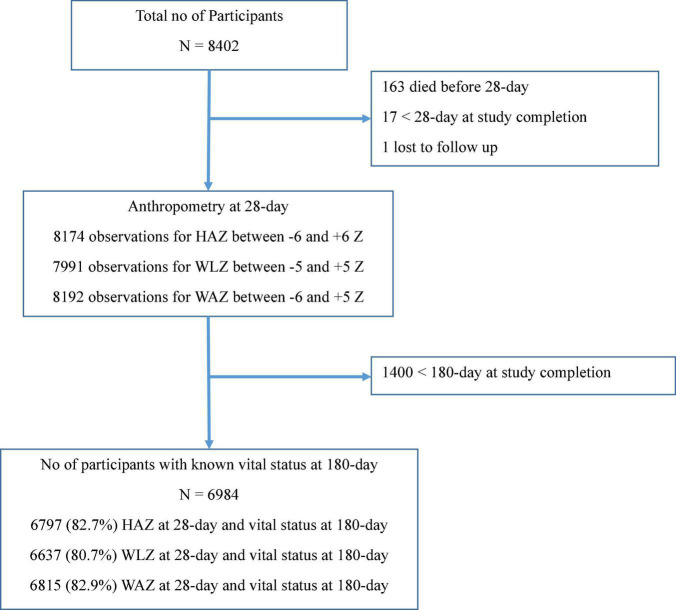
Flowchart showing the number of infants included in the analysis.

The primary trial included 8,402 LBW infants. The valid anthropometry data at 28-day and vital status at 180-day were available for 6,797 (82.7%), 6,637 (80.7%), and 6,815 (82.9%) infants for HAZ, WLZ, and WAZ, respectively, as shown in Figure 1.

Mean birthweight (*SD*) of the infants was 2,069 (168) g. The Mean (*SD*) weight and length at 28-day of age was 2.9 kg (0.4) and 49.2 cm (1.9), respectively. Mean (*SD*) of the WLZ, LAZ, and WAZ score was –1.03 (1.06), –2.49 (0.97), and –2.71 (0.92), respectively. Underweight (76.6%) was the most common anthropometric deficit followed by stunting (67.1%) and severe underweight (32.9%) as shown in Table 1.

**TABLE 1:** Anthropometric status at 28-day of age*.

Variables	At 1 month
Weight (kg), mean (SD)	2.93 (0.41)
Length (cm), mean (SD)	49.21 (1.91)
WLZ, mean (SD)	−1.03 (1.06)
LAZ, mean (SD)	−2.49 (0.97)
WAZ, mean (SD)	−2.71 (0.92)
Wasted, n (%)	1,123 (16.9)
Stunted, n (%)	4,562 (67.1)
Underweight, n (%)	5,221 (76.6)
Wasted and stunted, n (%)	699 (10.5)
Wasted and underweight, n (%)	1,076 (16.2)
Stunted and underweight, n (%)	4,094 (60.2)
Severely wasted, n (%)	289 (4.4)
Severely stunted, n (%)	1,840 (27.1)
Severely underweight, n (%)	2,248 (32.9)
Severely wasted and severely stunted, n (%)	120 (1.8)
Severely wasted and severely underweight, n (%)	261 (3.9)
Severely stunted and severely underweight, n (%)	1,432 (21.1)

*The number of observations available for HAZ, WLZ, and WAZ at 28-day were 6,797, 6,637, and 6,815, respectively.

Sensitivity, specificity, positive, and negative likelihood ratio, positive, and negative predictive values, AUC for different anthropometric indicators are presented in Table 2. Sensitivity was highest for WAZ < –2 (94.2%), followed by LAZ < –2 (90.7%) and WAZ < –3 (75.0%). Specificity was highest for WLZ < –2 (83.6%), followed by LAZ < –3 (73.6%) and WAZ < –3 (68.0%). Overall, among individual measures AUC was maximum for WAZ < –3 (0.72). Among composite measures, AUC was similar for all combinations at the cut-off of -2 (0.66). Concurrent severe underweight and severe stunting had the highest AUC at the cut-off of –3 (0.67).

**TABLE 2:** Sensitivity, specificity, positive and negative likelihood ratios, positive, and negative predictive values for cut-off point of anthropometry at 1 month of age and mortality between 1 and 6 months of age.

Anthropometric measure	Sensitivity^@^%	Specificity^#^%	LR^$^ +	LR^&^ –	PPV*	NPV^	AUC
Wasted	43.4	83.6	2.65	0.68	5.0	98.7	0.65
Stunted	90.7	33.4	1.36	0.28	3.0	99.4	0.64
Underweight	94.2	23.8	1.24	0.24	2.8	99.4	0.61
Wasted and stunted	38.8	90.0	3.88	0.68	7.2	98.7	0.67
Wasted and underweight	42.6	84.3	2.71	0.68	5.1	98.7	0.65
Stunted and underweight	88.7	40.4	1.49	0.28	3.3	99.4	0.66
Severely wasted	61.2	50.6	1.24	0.77	2.4	98.5	0.59
Severely stunted	55.6	73.6	2.11	0.60	4.6	98.7	0.66
Severely underweight	75.0	68.0	2.34	0.37	5.2	99.2	0.72
Severely wasted and severely stunted	60.5	51.8	1.26	0.76	2.4	98.5	0.58
Severely wasted and severely underweight	61.2	50.8	1.24	0.76	2.4	98.5	0.59
Severely stunted and severely underweight	52.3	79.6	2.56	0.6	5.5	98.7	0.68

LR, likelihood ratio; PPV, positive predictive value; NPV, negative predictive value; AUC, area under curve; LAZ, length-for-age z-score; WAZ, weight-for-age z-score; WLZ, weight-for-length z-score. ^@^ Proportion of infants having z-scores < –2 or < –3 at 1 month of age among those who died between 1 and 6 months of age; ^#^ Proportion of infants having z-scores ≥ –2 or ≥ –3 at 1 month of age among those who survived between 1 and 6 months of age. ^$^ Sensitivity/(1-Specificity); ^&^ (1-Sensitivity)/Specificity. * Proportion of infants who died between 1 and 6 months of age among those had z-scores < –2 or < – 3 at 1 month of age; ^ Proportion of infants who survived between 1 and 6 months of age among those had z-scores ≥ –2 or ≥ –3 at 1 month of age.

Stunting (4.81, 95% *CI* 2.77–8.36), wasting (3.78, 95% *CI* 2.65–5.38), and underweight (4.97, 95% *CI* 2.53–9.77) at 28-day were associated with higher risk of deaths between 28-day and 180-day of life compared to infants who were not stunted, wasted, and underweight, respectively. The risk of mortality was highest for concurrent severe wasting and severe stunting (6.90, 95% *CI* 3.94–12.08) although the PAF (0.10) was low. We found that severe underweight infants had higher risk (6.18, 95% *CI* 4.29–8.90) of mortality, the PAF was 0.63, and had the highest Harrell’s C concordance of 0.72 (Table 3).

**TABLE 3:** Month 1 anthropometry and child mortality (*n* = 170) from 1 to 6 months of age.

Cut-off point	Number	Death	Crude HR (95% CI)	Adjusted HR^#^ (95% CI)	PAF (95% CI)	Harrell’s C concordance
Wasted	1,123	56	3.84 (2.70 – 5.46) *	3.78 (2.65 – 5.38) *	0.32 (0.21 – 0.42)	0.65
Stunted	4,558	137	4.85 (2.79 – 8.42) *	4.81 (2.77 – 8.36) *	0.72 (0.54 – 0.83)	0.63
Underweight	5,217	147	5.03 (2.56 – 9.88) *	4.97 (2.53 – 9.77) *	0.75 (0.53 – 0.87)	0.61
Wasted and stunted	699	50	5.54 (3.87 – 7.94) *	5.45 (3.80 – 7.84) *	0.32 (0.22 – 0.41)	0.66
Wasted and underweight	1,076	55	3.92 (2.75 – 5.57) *	3.85 (2.70 – 5.50) *	0.32 (0.21 – 0.42)	0.65
Stunted and underweight	4,091	134	5.26 (3.17 – 8.74) *	5.23 (3.15 – 8.68) *	0.72 (0.56 – 0.82)	0.66
Severely wasted	289	20	4.13 (2.58 – 6.60) *	4.05 (2.53 – 6.49) *	0.12 (0.05 – 0.18)	0.58
Severely stunted	1,839	84	3.44 (2.47 – 4.78) *	3.42 (2.46 – 4.76) *	0.40 (0.27 – 0.50)	0.66
Severely underweight	2,247	117	6.23 (4.32 – 8.99) *	6.18 (4.29 – 8.90) *	0.63 (0.52 – 0.72)	0.72
Severely wasted and severely stunted	120	14	7.01 (4.02 – 12.23) *	6.90 (3.94 – 12.08) *	0.10 (0.04 – 0.15)	0.58
Severely wasted and severely underweight	261	20	4.61 (2.88 – 7.37) *	4.52 (2.82 – 7.24) *	0.12 (0.06 – 0.19)	0.59
Severely stunted and severely underweight	1,431	79	4.20 (3.02 – 5.84) *	4.17 (3.00 – 5.80) *	0.40 (0.29 – 0.50)	0.67

CI, confidence interval; PAF, population attributable fraction; LAZ, length-for-age; WAZ, weight-for-age; WLZ, weight-for-length. ^#^Adjusted for intervention. *Significant at p < 0.001.

## Discussion

Using data from a large individually controlled trial, we found that WAZ < –3 *SD* (severe underweight) measured at 28-day is the best predictor of mortality between 28-day and 180-day of life among LBW Indian children. Combination of different anthropometric measures did not perform better than individual measures. The hazard of death between 28-day and 180-day of life was higher among malnourished children for all anthropometric indicators.

Although evidence specific to LBW infants in the first 6 months of life is scarce, secondary analysis of data from India has also shown that WAZ < –3 *SD* was a better predictor of mortality during infancy (30). Information on birthweight was not available in this study (31). A recent systematic review of studies done, mostly from sub-Saharan Africa concluded that WAZ was better at identifying infants at risk of mortality/morbidity in the first 6 months of life (21).

The previous studies have shown that length measurement in field settings, especially during the first year of life is error prone (19). Additionally, WLZ cannot be assessed for all LBW infants as the same is defined only for ≥ 45 cm (25). Although LBW is a known risk factor for morbidity and mortality, our findings support identification of malnutrition in these infants to differentiate infants born LBW and growing normally requiring no intervention, from those malnourished and at higher risk of death requiring timely intervention. Supporting severely underweight LBW infants can potentially reduce mortality in this group by 63% (95% *CI* 52–72%). Although the risk of mortality was highest for concurrent severe wasting and severe stunting similar to previous studies, the population attributable fraction was low (16, 32, 33).

Weight estimation is easier, less error prone in field setting and is already integrated in the current national nutritional program in India (ICDS) for monitoring growth (34). The Home-Based Care for Young Children (HBYC) program currently includes additional visits for LBW infants. Using weight to track growth and nutritional status and identifying vulnerable infants will help streamline the ICDS and HBYC program (35). The potential challenge of having different anthropometric screening criteria for < 6 and 6–59 months can be overcome by rigorous training of frontline health workers in the assessment of both weight and length (30). Stunting, wasting, and underweight capture different aspects of growth in the same child. All three measures should be used concurrently, and not in isolation, to ensure survival and thrive of under five children. Since WAZ will identify a higher number of children as malnourished simultaneous strengthening of the health system in terms of inpatient and outpatient services for infants identified as SAM is necessitated (30).

To our knowledge, this is the first description of mortality in relation to anthropometric indicators exclusively among LBW infants. We used data from a large randomized controlled trial with minimal loss to follow up (31). Weight and length assessment was done by a standardized team and individual as well as combinations of anthropometric measures were used. Children with gross congenital malformations were excluded from the study and hence most of extreme wasting or underweight would be nutritional in origin. Our findings should be interpreted knowing that we used anthropometric indicators assessed at 28-day and hence we cannot comment on prediction of mortality in the neonatal period. Our sample did not include all LBW infants. The study setting had high burden of LBW and findings might differ in setting with lower prevalence. We could not analyze the predictive ability for specific causes of death as the same was not available in the data and the primary trial was powered to assess all-cause mortality.

## Conclusion

Severe underweight (WAZ < –3) better discriminated deaths among LBW infants < 6 months of age. It can be considered for diagnosis of nutritionally at-risk infants in this age group.

## Data Availability Statement

The dataset pertaining to the results reported in the manuscript will be made available to others only for health and medical research, subject to constraints of the consent under which the data was collected. De-identified individual participant data will be made available. Data will be available beginning 6 months and ending 5 years after publication of this article. Requests for data should be made to TC, tarun.choudhary@uib.no. A data sharing agreement that meets the data sharing requirements of the Society for Applied Studies (New Delhi, India) and Centre for International Health, University of Bergen (Norway) will be signed with the data requester.

## Ethics Statement

The study involved human participants. Written informed consent was obtained from the infant’s parents at enrollment. The primary trial was approved by the Ethics Committee of Society for Applied Studies in India, the Regional Committee for Medical and Health Research Ethics in Norway, and World Health Organization, Geneva.

## Author Contributions

TC: conceptualization, data acquisition, data analysis and interpretation, writing the first draft, manuscript editing, and finalization. BS, SS, and MK: data analysis and interpretation, manuscript editing, and finalization. SM, ST, and NB: interpretation of findings, critical revisions of the manuscript, and finalization. All authors read and approved the final manuscript.

## Conflict of Interest

The authors declare that the research was conducted in the absence of any commercial or financial relationships that could be construed as a potential conflict of interest.

## Publisher’s Note

All claims expressed in this article are solely those of the authors and do not necessarily represent those of their affiliated organizations, or those of the publisher, the editors and the reviewers. Any product that may be evaluated in this article, or claim that may be made by its manufacturer, is not guaranteed or endorsed by the publisher.

## Abbreviations

LBW: low birthweight
KMC: kangaroo mother care
WLZ: weight-for-length z-score
LAZ: length-for-age z-score
WAZ: weight-for-age z-score
AUC: area under receiver operating characteristics curves
MUAC: mid-upper-arm circumference
LMIC: lower- and middle-income countries
SD: standard deviation.
